# Generation and characterization of epoxide hydrolase 3 (*EPHX3*)-deficient mice

**DOI:** 10.1371/journal.pone.0175348

**Published:** 2017-04-06

**Authors:** Samantha L. Hoopes, Artiom Gruzdev, Matthew L. Edin, Joan P. Graves, J. Alyce Bradbury, Gordon P. Flake, Fred B. Lih, Laura M. DeGraff, Darryl C. Zeldin

**Affiliations:** Division of Intramural Research, National Institute of Environmental Health Sciences, Research Triangle Park, Durham, NC, United States of America; Institute of Neurology (Edinger-Institute), GERMANY

## Abstract

Cytochrome P450 (CYP) epoxygenases metabolize arachidonic acid into epoxyeicosatrienoic acids (EETs), which play an important role in blood pressure regulation, protection against ischemia-reperfusion injury, angiogenesis, and inflammation. Epoxide hydrolases metabolize EETs to their corresponding diols (dihydroxyeicosatrienoic acids; DHETs) which are biologically less active. Microsomal epoxide hydrolase (EPHX1, mEH) and soluble epoxide hydrolase (EPHX2, sEH) were identified >30 years ago and are capable of hydrolyzing EETs to DHETs. A novel epoxide hydrolase, EPHX3, was recently identified by sequence homology and also exhibits epoxide hydrolase activity *in vitro* with a substrate preference for 9,10-epoxyoctadecamonoenoic acid (EpOME) and 11,12-EET. EPHX3 is highly expressed in the skin, lung, stomach, esophagus, and tongue; however, its endogenous function is unknown. Therefore, we investigated the impact of genetic disruption of *Ephx3* on fatty acid epoxide hydrolysis and EET-related physiology in mice. *Ephx3*^*-/-*^ mice were generated by excising the promoter and first four exons of the *Ephx3* gene using Cre-*LoxP* methodology. LC-MS/MS analysis of *Ephx3*^*-/-*^ heart, lung, and skin lysates revealed no differences in endogenous epoxide:diol ratios compared to wild type (WT). *Ephx3*^*-/-*^ mice also exhibited no change in plasma levels of fatty acid epoxides and diols relative to WT. Incubations of cytosolic and microsomal fractions prepared from *Ephx3*^-/-^ and WT stomach, lung, and skin with synthetic 8,9-EET, 11,12-EET, and 9,10-EpOME revealed no significant differences in rates of fatty acid diol formation between the genotypes. *Ephx3*^*-/-*^ hearts had similar functional recovery compared to WT hearts following ischemia/reperfusion injury. Following intranasal lipopolysaccharide (LPS) exposure, *Ephx3*^*-/-*^ mice were not different from WT in terms of lung histology, bronchoalveolar lavage fluid cell counts, or fatty acid epoxide and diol levels. We conclude that genetic disruption of *Ephx3* does not result in an overt phenotype and has no significant effects on the metabolism of EETs or EpOMEs *in vivo*.

## Introduction

Epoxide hydrolases are involved in xenobiotic detoxification as well as metabolism of lipid signaling molecules. These enzymes belong to the structural family of α/β hydrolase fold enzymes [1] and hydrolyze epoxygenated compounds to their corresponding diols, which often have less biological activity than the parent epoxide. Cytochrome P450 (CYP) epoxygenases metabolize arachidonic acid (AA) to epoxyeicosatrienoic acids (EETs). Similarly, CYP epoxygenases metabolize linoleic acid (LA) and the omega-3 fatty acids, eicosapentaenoic acid (EPA) and docosahexaenoic acid (DHA), to epoxides. [2]. EETs have potent angiogenic [3,4], cardioprotective [5,6], anti-inflammatory [7], and vasodilatory effects [8,9]. Soluble epoxide hydrolase (sEH or EPHX2) is the primary enzyme responsible for EET metabolism, converting them to dihydroxyeciosatrienoic acids (DHETS) [10]. Genetic disruption or pharmacological inhibition of EPHX2 impacts hypertension, ischemic heart disease, sepsis, and atherosclerosis in animal models [11]. Due to low catalytic turnover, microsomal epoxide hydrolase (mEH or EPHX1) is not thought to play a significant role in EET hydrolysis *in vivo* [12].

Recently, a novel epoxide hydrolase, EPHX3, was identified by the presence of a highly conserved 16 amino acid motif found in other epoxide hydrolase-related α/β hydrolase fold enzymes [12]. EPHX3, along with another recently identified candidate epoxide hydrolase, EPHX4, have 45% sequence homology to each other and are most closely related to a group of epoxide hydrolases from *Caenorhabditis elegans* [12,13]. Mammalian EPHX3 was originally termed Abhydrolase Domain-Containing Protein 9 (ABHD9), but was renamed following *in vitro* studies that showed epoxide hydrolase activity against EETs and epoxyeicosamonoenoic acids (EpOMEs) [12]. Recombinant EPHX3 exhibited the highest catalytic efficiency for hydrolysis of 9,10-EpOME followed by 11,12-EET, 14,15-EET, and 8,9-EET [12]. Interestingly, EPHX3 hydrolyzed substrates with a high *V*_*max*_ and a high *K*_*m*_ suggesting catalytic efficiencies similar to that observed for both EPHX1 and EPHX2 [12].

Previous studies have shown that the *Ephx3* gene is highly expressed in lung, skin, stomach, tongue, and esophagus in wild type (WT) mice [12]. This is a significantly different expression pattern than both *Ephx1* and *Ephx2* genes. Previous studies have also shown that EPHX2 is responsible for the majority of EET metabolism; however, genetic disruption or pharmacological inhibition of EPHX2 does not completely eliminate diol formation suggesting that other enzymes may also contribute to EET hydrolysis [14,15]. Since there are no known EPHX3-selective inhibitors, we generated *Ephx3*^*-/-*^ mice to investigate the role of EPHX3 as an epoxide hydrolase *in vivo*. Our findings indicate that EPHX3 does not play a significant role in the metabolism of fatty acid epoxides *in vivo*. Thus, while EPHX3 demonstrates fatty acid epoxide hydrolase activity *in vitro*, it does not have a similar function *in vivo* under basal conditions. Moreover, *Ephx3*^-/-^ mice do not exhibit an overt phenotype in models where EETs are known to play a biological role, including cardiac ischemia-reperfusion injury and LPS-induced lung inflammation.

## Materials and methods

### Generation of EPHX3-deficient Mice

**Fig 1:**
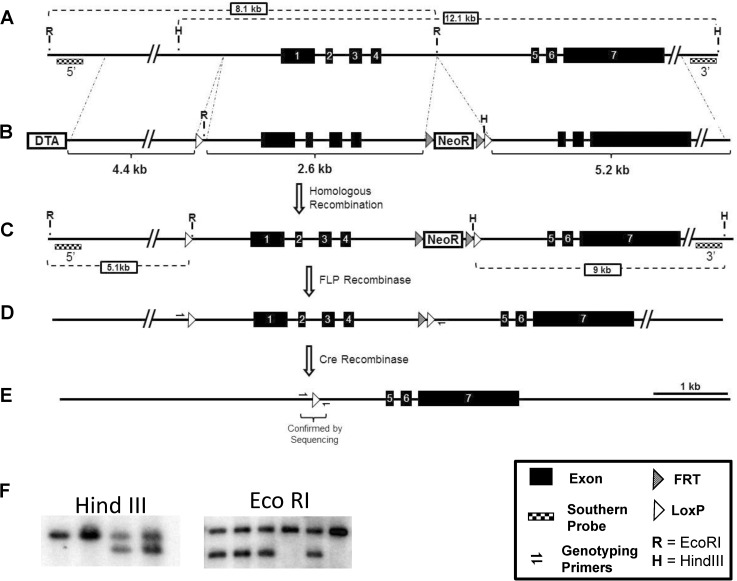
Generation of *Ephx3*^-/-^ mice. A) Wild type *Ephx3* locus on the reverse strand of chromosome 17:b1. B) Targeting vector to introduce *LoxP* sites flanking the promoter region and first four exons of *Ephx3*. Targeting vector constructed with *FRT*-site flanked neomycin resistance positive selection and MC1-DTA negative selection. C) Targeted *Ephx3* locus following homologous recombination. D) *Ephx3* “flox” locus following excision of neomycin resistance cassette via *in vivo* FLP recombinase exposure. E) *Ephx3* null allele generated by breeding to germline expressing Cre recombinase mouse line (Prm-Cre). F) Representative Southern blots using both the 5’ and 3’ probes to confirm proper recombination event. Only relevant restriction enzymes sites shown with expected fragment sizes.

The targeting scheme introduced a *LoxP* site at position -506 relative to the transcription start site and a *LoxP* site with a FRT-flanked Neomycin Resistance cassette 181 bp 3’ of exon 4. The final targeting vector construct contained (5’ to 3’) a 5.2 kb homology arm, a *LoxP site*, a 1975 bp replacement/middle arm, a Frt-PGK-NeoR-Frt cassette, a *LoxP* site, a 5.2 kb homology arm, and a MC1-DTA negative selection cassette (Fig 1). Successful targeting of 129S6 ES cells was confirmed by Southern blot analysis using an external and internal probe, generated by the following primer sets: E3pr5-Fwd: 5’-CTGGGAGTGGGGAGGTGGGG-3’; E3pr5-Rev: 5’-CAGTCCTCGTGGGGGAGGGG-3’, E3pr3-Fwd: 5’-ATGTCTGGTTTCTGCGATGCT-3’, E3pr3-REV: 5’-CTCCTGTGGTTACTGATGTCTTTC-3’. Germline transmitting chimeras were bred to 129S4-Gt(ROSA)26Sor^tm1(FLP1)Dym^ mice to excise the Frt-flanked NeoR cassette and generate the conditional-null *Ephx3* allele (*Ephx3* flox allele). Genotyping of the flox and WT alleles was done with the following primers that yield a 471 bp amplicon from the flox allele and 545 bp amplicon from the WT allele: E3Common: 5’-GACCTGGCACGGGGATCCAGA-‘3; E3Flox: 5’-GGCGTATCACGAGGCCCTTTCG-3’; and E3WT: 5’- CCCCTGCCCCTCAAGTGCTGA-3’. The *Ephx3*-null allele was generated by breeding the 129S-Ephx3^fx/fx^ mice to 129S4-tg(Prm-cre)580g mice. The male progeny of this cross express Cre recombinase in their germline and transmit the Cre-recombined *Ephx3*-null allele to their offspring. The *Ephx3*^+/-^ tg(Cre) negative mice were in-crossed to generate *Ephx3*^-/-^ and WT littermate controls on a pure 129S genetic background. Genotyping of the null allele was done with the following primers that yield a 114 bp amplicon from the null allele: E3Flox (sequence above) and E3Null: 5’- GGCCACTAGCAAGCTTACCTA-3’. Proper germline Flp and Cre-mediated recombination of the targeted locus was confirmed by Southern blot analysis in Flp-negative and Cre-negative mice.

### Animals

Body and organ weights were recorded in age- and sex-matched littermates (8–10 weeks old). Mice were euthanized with a lethal dose of pentobarbital and tissues were collected and either frozen in liquid nitrogen or used immediately. All animal experiments were performed according to NIH guidelines and were approved by the National Institute of Environmental Health Sciences Animal Care and Use Committee.

### Quantitative RT-PCR analysis

RNA was extracted from tissues using the PureLink Mini Kit (Ambion) following the manufacturer's protocol, and quantitated using a NanoDrop 8000 (Thermo Fisher Scientific). RNA was reverse transcribed using the High-Capacity cDNA Reverse Transcription Kit (Life Technologies) according to the manufacturer’s protocol. Expression of murine *Ephx1*, *Ephx2*, *Ephx3*, *Ephx4*, *Cyp2c37*, *Cyp2c39*, *Cyp2c40*, *Cyp2c44*, *Cyp2c50*, *Cyp2c54*, *Cyp2c55*, *Cyp2c65*, *Cyp2c66*, *Cyp2c70*, *Cyp2j6*, *Cyp2j9*, *Cyp2j11*, *Cyp2j12*, and *Gapdh* were quantified by quantitative RT-PCR (qPCR) using TaqMan Assays on Demand (Applied Biosystems) or SYBR Green (Thermo Fisher Scientific) as described previously [16]. Expression of all genes was normalized to *Gapdh* using the 2^−ΔΔ*Ct*^ method [17].

### Lysate incubations

Tissues were lysed with lysis buffer [10ml PBS + protease inhibitor cocktail tablets (cOmplete Mini: EDTA-free; Roche Diagnostics)] in a TissueLyser II (10 minutes at 30 Hz; Qiagen). Protein concentrations were determined by BCA assay. Lysates were diluted to 0.5 μg/μl with PBS. Eighty microliters of assay buffer (PBS+0.1mg/ml BSA) was added to 20μl of lysate and incubated with 8,9-EET, 11,12-EET, or 9,10-EpOME (0.8 µM) for 5 min at 37°C. A cold acid wash (500 μl 0.1% acetic acid in 5% methanol) was added to stop the reactions. Free lipids were extracted from samples using ethyl acetate, and then dried by vacuum centrifugation and reconstituted in 30% ethanol for LC-MS/MS analysis.

### Preparation of microsomal and cytosolic fractions

Tissues were homogenized in 1.5ml of a sucrose (0.25M)/Tris-Cl (10mM; ph7.5) solution with protease inhibitors including leupeptin (1μg/ml), aprotinin (1μg/ml), PMSF (0.25μM), and pepstatin (1μg/ml). Tissues were homogenized using a TissueLyser II (10 minutes at 30 Hz; Qiagen). Lysates were transferred to centrifuge tubes for a series of centrifugations (Centrifuge 5415D; Eppendorf). Samples were initially spun at 5,000 rpm for 20 minutes at 4°C. The supernatants were removed and spun twice at 12,000 rpm for 20 minutes at 4°C. The supernatants were then collected and spun at 100,000g using an ultracentrifuge (Beckman TL-100). The high speed supernatants were collected as the cytosolic fractions and stored at -80°C. The microsomal fractions (high speed pellets) were resuspended in a solution of 50mM Tris (pH 7.5), 1mM DTT, 1mM EDTA, and 20% glycerol using a Teflon homogenizer at 40% max speed on ice, and frozen at -80°C until use. A total of 25μl of resuspension buffer was used for skin samples and 50μl was used for lung and stomach samples. As with the lysate incubations described above, 20 μl microsomal and cytosolic fractions were incubated for 5 minutes with synthetic 11,12-EET (0.8 μM) with/without the EPHX2 inhibitor t-AUCB (1.0 μM). DHET formation was determined by LC-MS/MS.

### LC-MS/MS

Free oxylipids were extracted from homogenized tissues (20 mg), plasma, and microsome/cytosol incubations. For the tissue lysate and incubation samples, an acid wash (500 μl 0.1% acetic acid in 5% methanol) was added to the samples which were then transferred to glass tubes containing ethyl acetate and 10 μl of internal standards (300 pg/ml PGE_2_-d_4_, 10,11-DiHN, and 10,11-EpHep; Cayman). Two sequential liquid:liquid extractions with 2 ml ethyl acetate were performed and the ethyl acetate layer was transferred to glass tubes containing 6 μl 30% glycerol. The ethyl acetate was evaporated, layered with argon, and stored at −80°C until analysis. Extraction of oxylipids from plasma was performed as described above with a few modifications. Only 250 μl acid methanol was added to the samples. After the ethyl acetate was evaporated from the samples, they were re-suspended in 1ml of 80% methanol and passed through Phree Phospholipid removal columns (Phenomenex). An additional 1ml of 80% methanol wash was used to transfer remaining sample to the column. The 80% methanol was evaporated, and then samples were layered with argon and stored at −80°C until analysis. Samples were reconstituted in 50 μl of 30% ethanol and injected into an Agilent 1200 series capillary HPLC (Agilent Technologies, Santa Clara, CA, USA) coupled to an API 3000 triple quadrupole mass spectrometer (PE Sciex) as described previously [18]. Quantitation of eicosanoids was performed using the Analyst 1.51 software (SCIEX), and levels were normalized to protein concentrations. Samples were analyzed in duplicate or triplicate.

### Mouse inflammation model

Mice (10–12 weeks old) were administered intranasal LPS (50 μg in 75μl saline) or saline alone (control). After 4 hours, the lungs were lavaged with 2 x 1ml Hank's buffered saline solution. Total cell counts in the bronchoalveolar lavage fluid (BALF) and cell differentials were determined using the Coulter particle counter. Cell differentials were analyzed by researchers blinded to genotype groups. Lungs were fixed in 4.0% paraformaldehyde, embedded in paraffin, sectioned (5μm), and stained with hematoxylin and eosin. Lung sections were scored by a board-certified pathologist blinded to genotype using an Olympus BX51 microscope. The histopathologic reaction to LPS was graded on the basis of four parameters: 1) margination of neutrophils on the vascular endothelium (0–4+); 2) perivascular neutrophilic infiltrate (0–4+); 3) peribronchial neutrophilic infiltrate (0–4+); and 4) perivascular and peribronchial hemorrhage (0–4+). Grading of each parameter was based upon both intensity and extent (the number of vessels or bronchi involved). The grades for each parameter were then combined to determine a total score for each animal, ranging from 0–16.

### Mouse ischemia/Reperfusion model

Hearts were perfused in a retrograde fashion using the Langendorff apparatus as previously described [5], with modified Krebs–Henseleit buffer (120 mM NaCl, 25 mM NaHCO_3_, 4.7 mM KCl, 1.2 mM KH_2_PO_4_, 1.2 mM MgSO_4_, 11 mM glucose, and 1.8 mM CaCl_2_) aerated with 95% air and 5% CO_2_. Following 40 min of stabilization, hearts were subjected to 20 min of global no-flow ischemia, followed by 40 min of reperfusion. Recovery of contractile function was expressed as the percentage of left ventricular developed pressure (LVDP) after 40 min of reperfusion relative to pre-ischemic LVDP. Rate pressure product (RPP) was calculated as LVDP × heart rate. Hearts were then frozen for RNA isolation.

### Statistical analysis

Values are expressed as means ± SEM. Two-tailed analyses were performed using a Student's *t* test with Microsoft Excel and GraphPad Prism. Values of *p*<0.05 were considered significantly different.

## Results

### Generation and initial characterization of *Ephx3*^*-/-*^ mice

**Fig 2:**
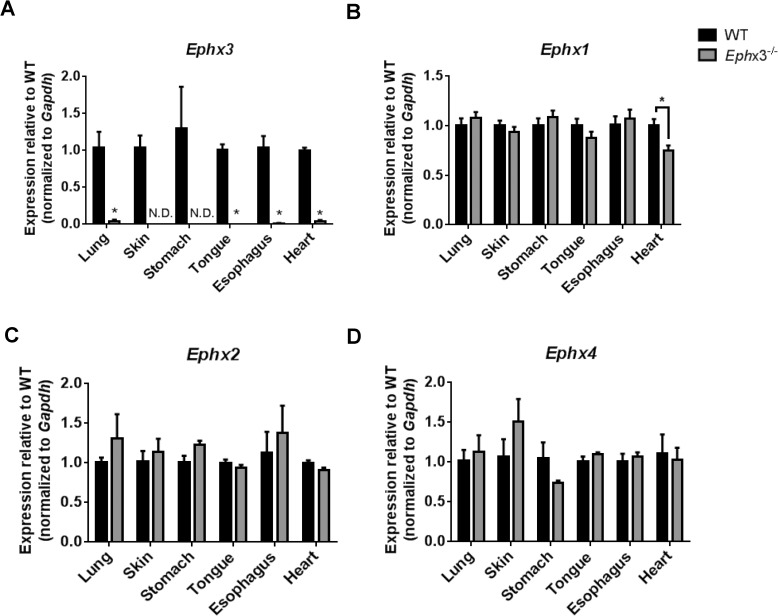
Expression of epoxide hydrolase isoforms in *Ephx3*^*-/-*^ mice. Quantitative RT-PCR of (A) *Ephx3*, (B) *Ephx1*, (C) *Ephx2*, and (D) *Ephx4* in *Ephx3*^*-/-*^ and WT mice. (*p<0.05; n = 3–4).

**Fig 3:**
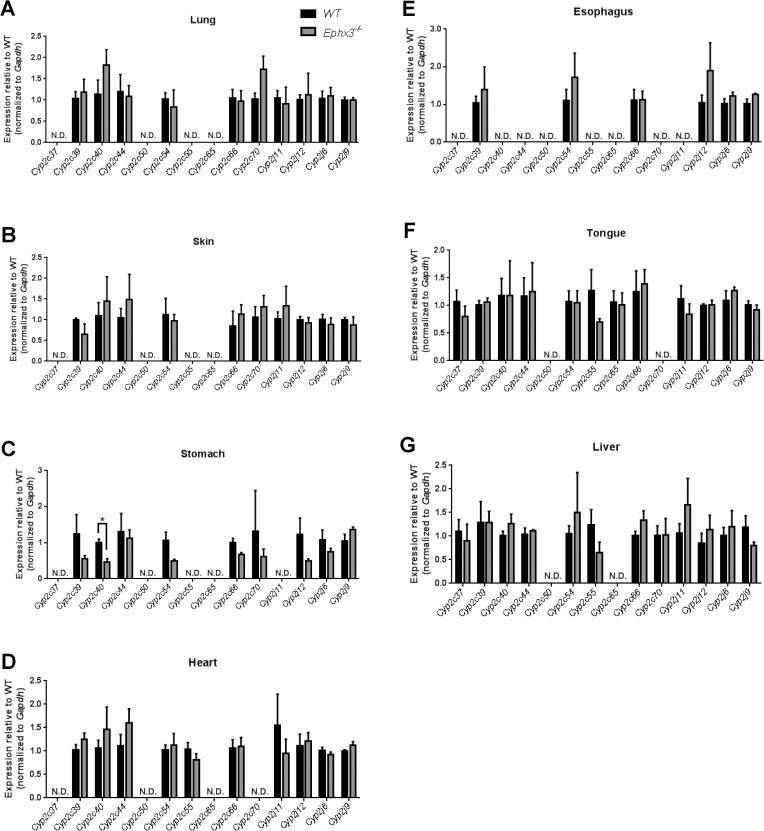
Disruption of *Ephx3* does not alter mRNA levels of CYP epoxygenases. Quantitative RT-PCR of *Cyp2c* and *Cyp2j* isoforms in (A) lung, (B) skin, (C) stomach, (D) heart, (E) esophagus, (F) tongue, and (G) liver of *Ephx3*^*-/-*^ and WT mice. (*p<0.05; n = 3–5, p = NS).

Mice with global disruption of the *Ephx3* gene were generated using standard Cre-*LoxP* methodology which targeted the proximal promoter and first four exons of the gene (Fig 1A–1E). The first 4 exons contain the previously identified hydrolase domain, and encode 63% of the protein (270 of 425 AA). Proper targeting was confirmed by Southern blot analysis (Fig 1F). While there are no immunospecific antibodies to EPHX3, qPCR results showed that *Ephx3* mRNA levels were low/undetectable in various tissues from the *Ephx3*^*-/-*^ mice (Fig 2A). To determine if there were compensatory changes in other known epoxide hydrolases in *Ephx3*^-/-^ mice, qPCR analysis was also performed using specific primer/probe sets for *Ephx1*, *Ephx2*, and *Ephx4*. Levels of the other epoxide hydrolases were similar in *Ephx3*^*-/-*^ mice relative to WT controls (Fig 2B–2D). Cardiac *Ephx1* expression was significantly lower in *Ephx3*^-/-^ hearts relative to WT; however, *Ephx1* expression in the heart is relatively low in WT mice and the 20% decrease in *Ephx3*^-/-^ mice is unlikely to be physiologically relevant. We also examined expression of *Cyp2j and Cyp2c* genes in *Ephx3*^*-/-*^ and WT tissues by qPCR. Expression levels of these CYP epoxygenases were largely unaffected by *Ephx3* genetic disruption in the tissues analyzed (Fig 3A–3G).

### Disruption of Ephx3 does not affect body weight, organ weight, or reproductive capacity

**Fig 4:**
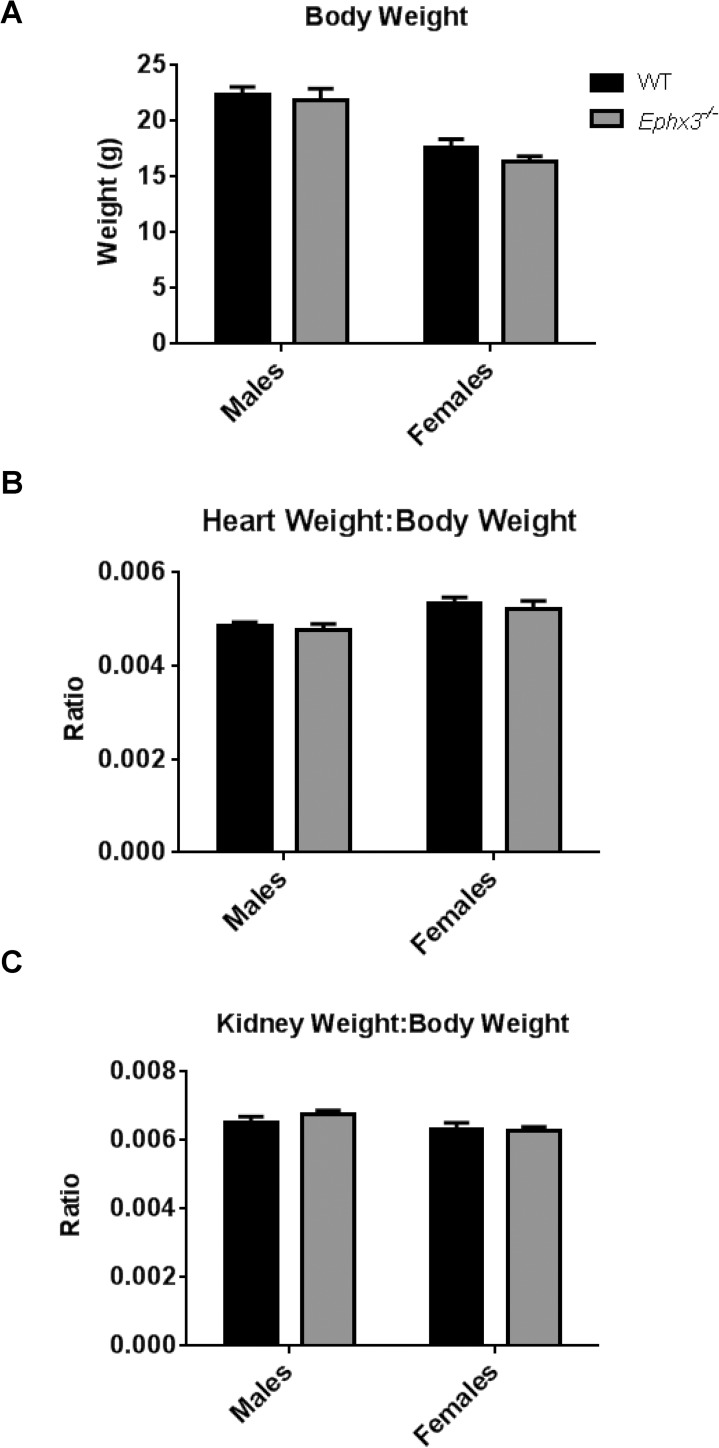
Body weight and organ weight:body weight ratios are not altered in *Ephx3*^*-/-*^ *mice*. (A) Body weight of both male and female WT and *Ephx3*^*-/-*^ mice. (B) Heart weight:body weight ratio and (C) kidney weight:body weight ratio in *Ephx3*^*-/-*^ and WT mice (n = 5–10, p = NS).

*Ephx3* disruption did not alter reproductive capacity. In-crossing of *Ephx3*^*+/-*^ mice resulted in litters that approximated Mendelian ratios. Of 223 heterozygous breeding litters analyzed, the average litter size was 5.5 with a male:female ratio of 624:598 (ratio male:female ratio = 1.04) and a genotype breakdown of 301(+/+):615(+/-):304(-/-) (ratio 1.0:2.0:1.0). Moreover, breeding *Ephx3*^*-/-*^ male to *Ephx3*^*-/-*^ female mice resulted in successful parturition, with an average litter size of 5.7 (14 litters analyzed). *Ephx3* disruption also did not alter body or organ weights. Thus, *Ephx3*^*-/-*^ mice and WT mice (8–10 weeks old) had similar body weights (Fig 4A), kidney weight:body weight ratios (Fig 4B) and heart weight:body weight ratios (Fig 4C).

### Endogenous epoxide:Diol ratios are unchanged in *Ephx3*^*-/-*^ mice under basal conditions

**Fig 5:**
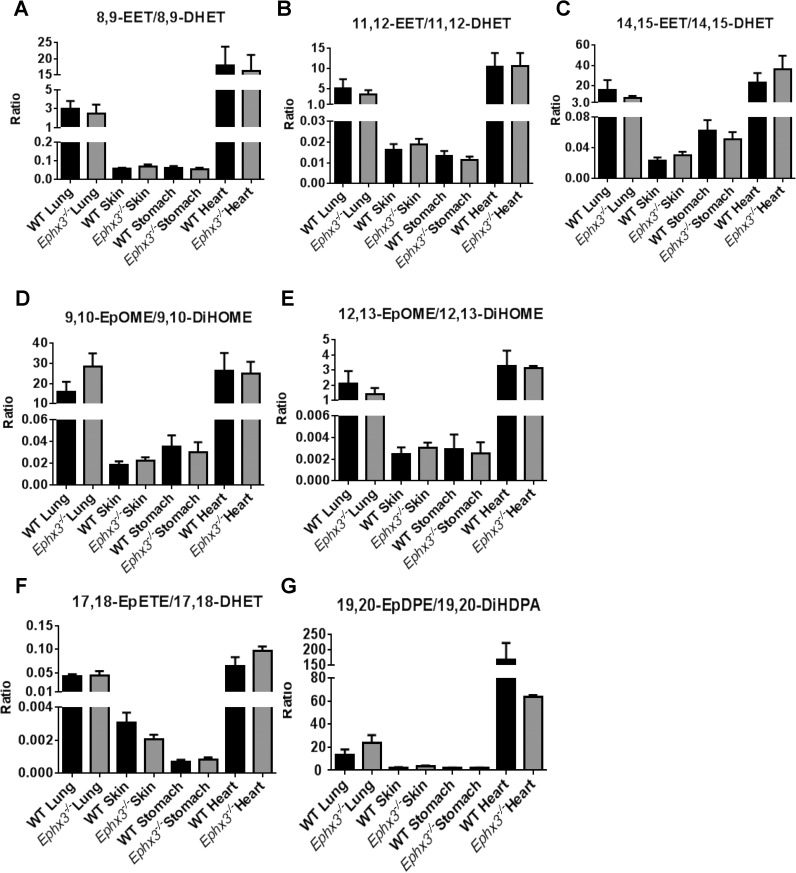
LC-MS/MS analysis shows no differences in endogenous epoxide:diol ratios in the *Ephx3*^*-/-*^ mice. Endogenous epoxide:diol ratios for (A) 8,9-EET/8,9-DHET, (B) 11,12-EET/11,12-DHET, (C) 14,15-EET/14,15-DHET, (D) 9,10-EpOME/9,10-DiHOME, (E) 12,13-EpOME/12,13-DiHOME, (F) 17,18-EpETE/17,18-DHET, and (G) 19,20-EpDPE/19,20-DiHDPA were determined in the heart, lung, skin, and plasma by LC-MS/MS in *Ephx3*^*-/-*^ and WT mice (n = 3–10, p = NS).

Previous *in vitro* studies showed that EPHX3 has epoxide hydrolase activity with high catalytic efficiency for 8,9-EET, 11,12-EET, and 9,10-EpOME. To determine if EPHX3 performs significant hydrolysis of fatty acid epoxides *in vivo*, LC-MS/MS analysis was performed on tissue lysates prepared from lung, skin, stomach, and heart samples. We quantified levels of arachidonic acid (Fig 5A–5C), linoleic acid (Fig 5D and 5E), EPA (Fig 5F), and DHA (Fig 5G)-derived epoxides and diols. There were no differences in epoxide:diol ratios between *Ephx3*^*-/-*^ and WT mice in any of these tissues.

### *Ephx3*^*-/-*^ mice have unchanged diol formation rates in vitro

**Fig 6:**
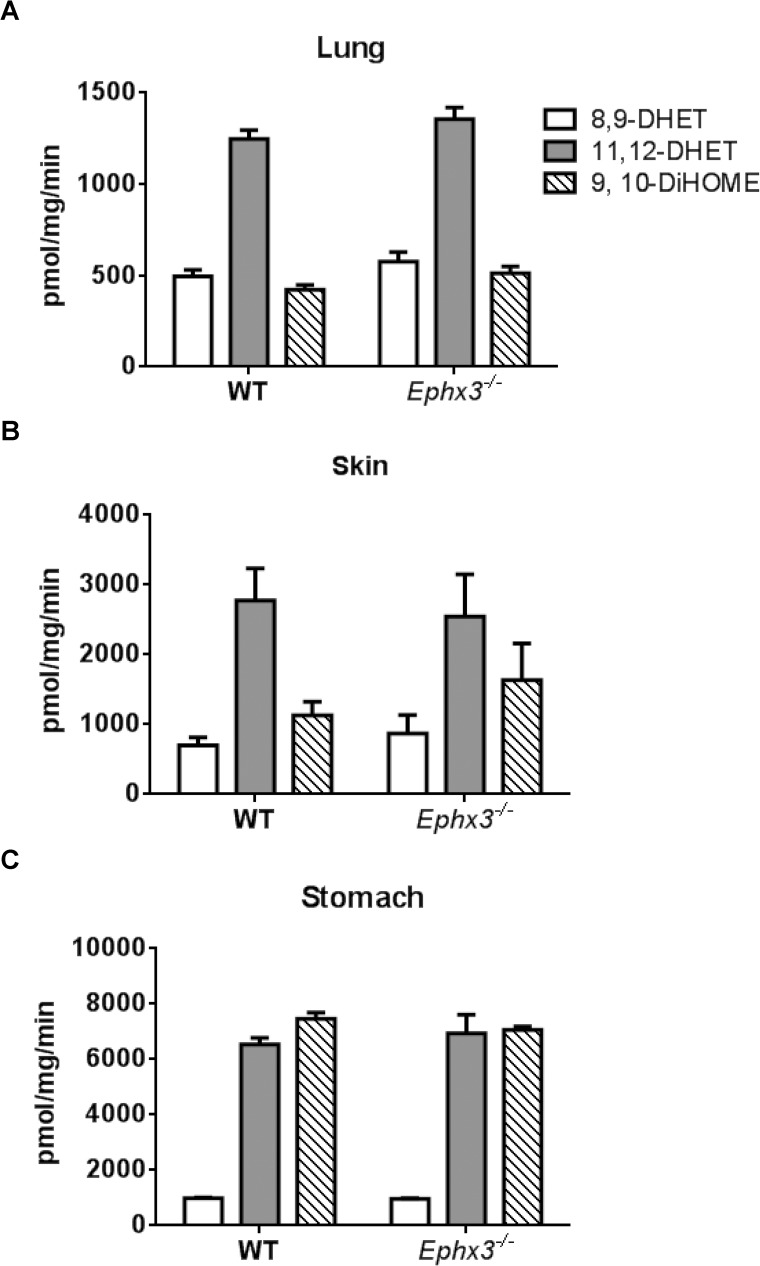
Incubation with synthetic EETs/EpOMEs showes no changes in diol formation rate in *Ephx3*^*-/-*^ mice. LC-MS/MS analysis of diol formation after a 5 minute incubation with synthetic EETs or EpOMEs in (A) lung, (B) skin, and (C) stomach lysates from *Ephx3*^*-/-*^ and WT mice. (n = 5–10, p = NS).

**Fig 7:**
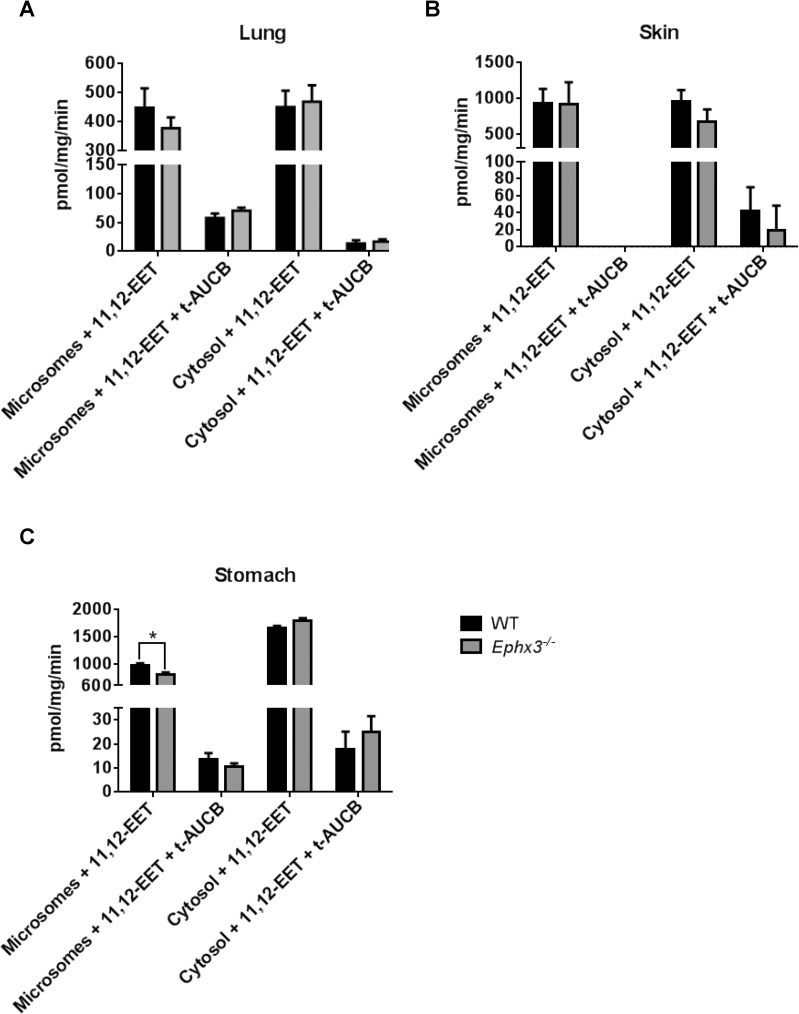
11,12-DHET formation rates are largely unchanged in microsomal and cytosolic fractions from various tissues of *Ephx3*^-/-^ mice. LC-MS/MS analysis of 11,12-DHET formation in microsomal and cytosolic fractions after a 5 minute incubation with synthetic 11,12-EET with/without the EPHX2 inhibitor t-AUCB in (A) lung, (B) skin, and (C) stomach samples (n = 3–6, *p<0.05).

Although no changes were observed in endogenous epoxide:diol ratios between genotypes; we investigated the potential role of EPHX3 in fatty acid epoxide hydrolysis by incubating tissue lysates with synthetic oxylipid substrates. Lung, skin, and stomach lysates were incubated with exogenous 8,9-EET, 11,12-EET, or 9,10-EpOME for 5 minutes. LC-MS/MS analysis showed that formation of 8,9-DHET, 11,12-DHET, and 9,10-EpOME was similar in lysates from *Ephx3*^*-/-*^ and WT mice (Fig 6A–6C). We also isolated microsomal and cytosolic fractions from lung, skin, and stomach samples and incubated with synthetic 11,12-EET. The 11,12-DHET formation rates were unchanged in both cytosolic and microsomal fractions from lung (Fig 7A), skin (Fig 7B), and stomach (Fig 7C). Co-incubation with the EPHX2 inhibitor t-AUCB, which has no inhibitory effect on recombinant EPHX3 *in vitro* [12], resulted in a significant decrease in diol formation; however, this decrease was of similar magnitude in cytosol and microsomal fractions from both genotypes (Fig 7A–7C).

### Inflammatory response to LPS is similar in *Ephx3*^*-/-*^ and WT mice

**Fig 8:**
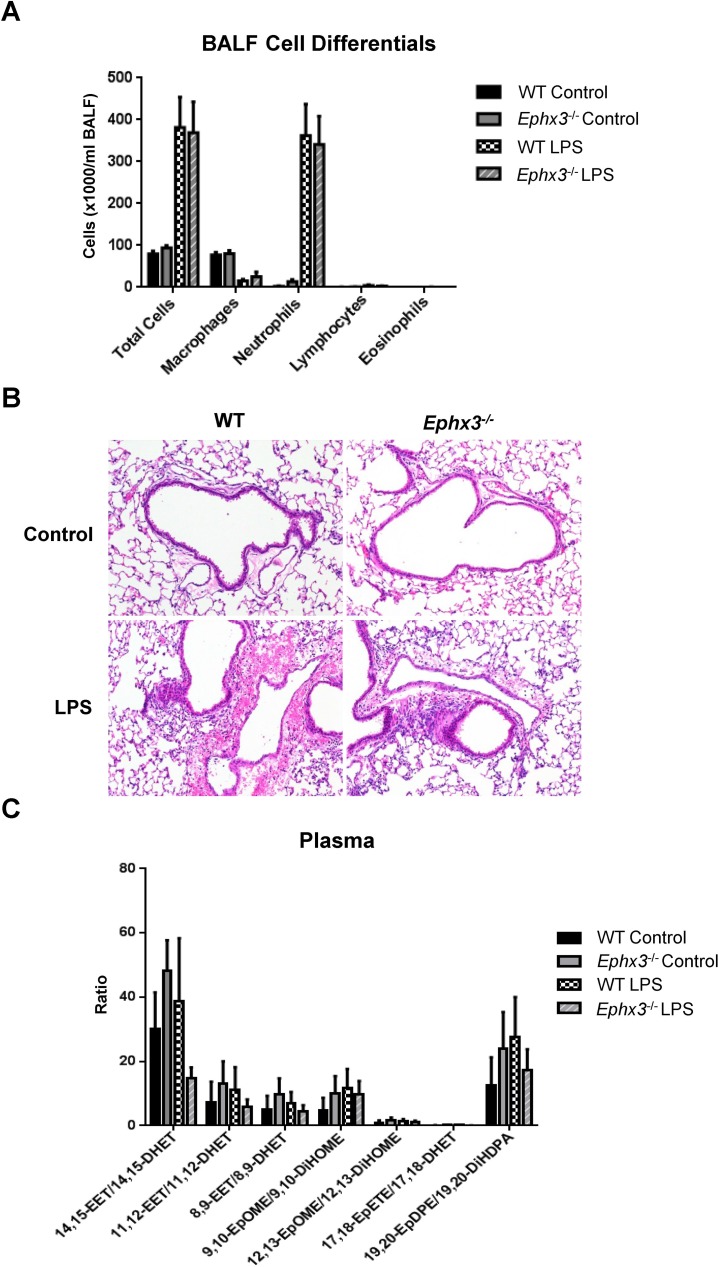
Bronchoalveolar lavage fluid cells, lung histology, and plasma epoxide:diol ratios are unchanged in *Ephx3*^*-/-*^ mice after LPS treatment. (A) Bronchoalveolar lavage fluid cell counts and differentials, (B) lung histology, and (C) LC-MS/MS epoxide/diol ratios in plasma from *Ephx3*^*-/-*^ and WT mice four hours after intranasal LPS exposure (n = 4–6; p = NS, original magnification = 10x).

The *Ephx3*^*-/-*^ mice did not exhibit an overt phenotype under basal conditions. To determine if an inflammatory challenge would unmask differences between the genotypes, mice were treated with LPS intranasally. Increased neutrophil numbers were observed in bronchoalveolar lavage fluid 4 hours after LPS exposure; however, the inflammatory response was similar in WT and *Ephx3*^*-/-*^ mice (Fig 8A). Moreover, lungs from WT and *Ephx3*^*-/-*^ mice exposed to LPS exhibited comparable levels of perivascular and peribronchial infiltration of neutrophils and extravasation of red blood cells into alveolar spaces. In contrast, lungs not exposed to LPS showed no inflammation or hemorrhage (Fig 8B; Table 1). After LPS stimulation, *Ephx3*^*-/-*^ mice were similar to WT in plasma epoxide:diol ratios and concentrations of prostaglandins and HETEs (Fig 8C, Table 2).

**Table 1:** Inflammation scores in LPS-induced lung injury model.

	Neutrophil margination	Perivascular neutrophils	Peribronchial neutrophils	Alveolar neutrophils	Perivascular/Peribronchial hemorrhage	Total score (0–16)
**WT Control**	0	0	0	0	0	0
**Ephx3**^**-/-**^ **Control**	0	0	0.2±0.2	0	0	0.2±0.2
**WT LPS**	1.3±0.3	1.6±0.4	2.1±0.3	1.1±0.3	0.3±0.2	5.3±0.8
**Ephx3**^**-/-**^ **LPS**	1.4±0.4	1.3±0.3	1.7±0.2	1.1±0.1	0.7±0.4	5.1±0.7

**Table 2:** Eicosanoids and Prostaglandins (pg/ml) in Plasma from *Ephx3*^*-/-*^ and WT mice treated with or without LPS.

	PGE_2_	PGD_2_	PGB_2_	PGF_2α_	12-HETE	19-HETE	20-HETE
**WT Control**	47±6	92±23	112±43	171±34	774±262	80±14	218±21
***Ephx3***^***-/-***^**Control**	38±5	93±32	219±142	92±21	626±227	75±16	213±46
**WT** **LPS**	66±16	112±24	195±53	172±49	596±279	75±17	424±35
***Ephx3***^***-/-***^**LPS**	63±19	144±17	160±52	188±40	714±254	75±31	417±60

### Post-ischemic cardiac functional recovery was not altered in the *Ephx3*^*-/-*^ mice

**Fig 9:**
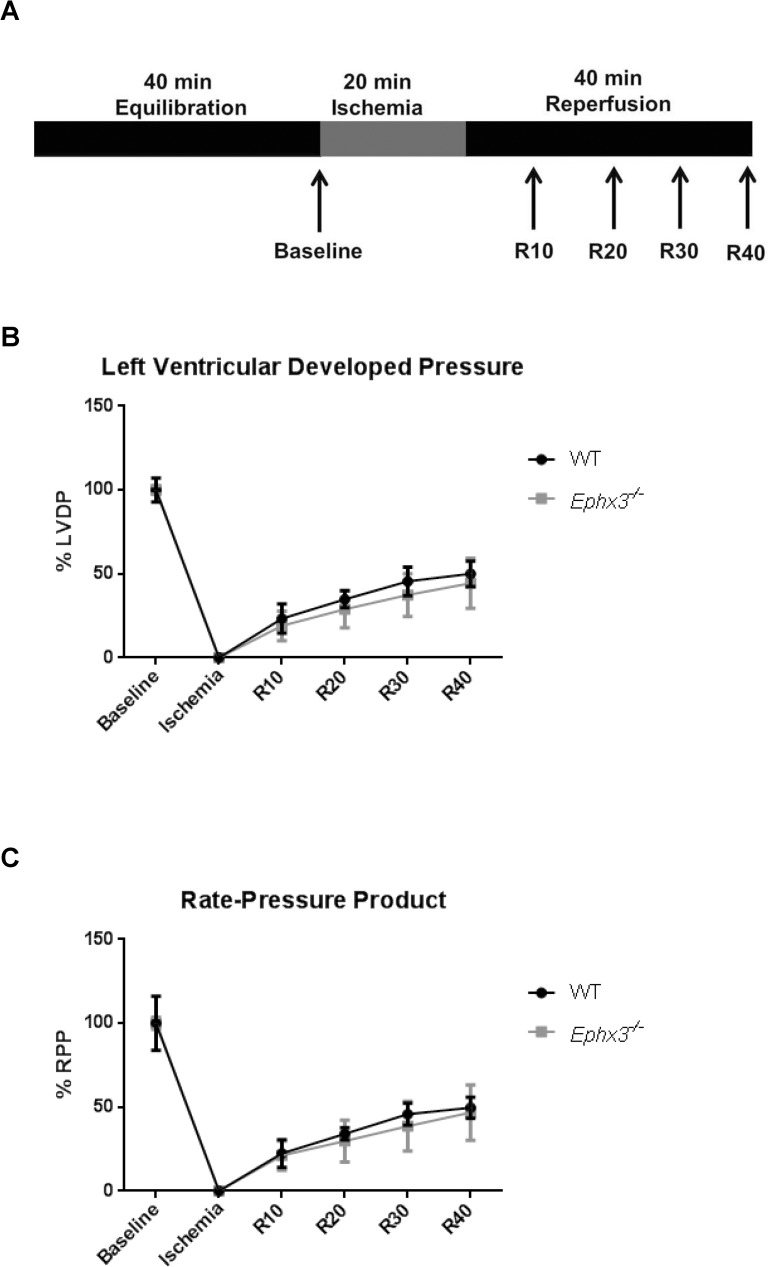
Cardiac recovery is unchanged in *Ephx3*^*-/-*^ hearts after global ischemia/reperfusion. (A) Hearts were allowed to equilibrate for 40 min, subjected to 20 min of global, no-flow ischemia, and then reperfused for 40 min using the Langendorff system. (B) Time course for recovery of left ventricular developed pressure. (C) Time course for recovery of rate pressure product (n = 3–4, p = NS).

Genetic disruption of EPHX2 results in increased epoxide:diol ratios and improves functional recovery following cardiac ischemia/reperfusion injury. Hearts isolated from WT and *Ephx3*^*-/-*^ mice were subjected to global no-flow ischemia followed by 40 minutes of reperfusion (Fig 9A). Left ventricular developed pressure, a measurement of cardiac function, was similar in *Ephx3*^*-/-*^ hearts compared to WT at baseline and following ischemia/reperfusion injury (Fig 9B). The rate pressure product, a surrogate measure of cardiac output, was also unchanged between genotypes (Fig 9C).

## Discussion

Epoxide hydrolases catalyze the hydrolysis of epoxygenated compounds to their corresponding vicinal diols. Oxylipids, such as EETs, regulate a wide variety of biologically processes including blood pressure, inflammation, and angiogenesis. Therefore, it is important to understand the enzymes involved in regulating steady-state levels of these metabolites. While EPHX2 has been shown to be responsible for the majority of EET metabolism *in vivo*, neither genetic disruption nor pharmacological inhibition of EPHX2 completely abolishes DHETs formation [14,15]. The contribution of the recently discovered EPHX3 to the metabolism of EETs is unknown. In this manuscript, we generated *Ephx3*^*-/-*^ mice which are viable and fertile. *Ephx3*^*-/-*^ mice develop normally and have no overt phenotype under basal conditions. Moreover, *Ephx3* disruption is not accompanied by compensatory changes in expression of CYP epoxygenases or other epoxide hydrolases. Importantly, *Ephx3* disruption does not alter fatty acid epoxide hydrolysis in vivo or in vitro. In addition, *Ephx3*^*-/-*^ mice do not exhibit altered responses in disease models known to be influenced by EETs including LPS-induced lung inflammation and post-ischemic cardiac dysfunction. While we did not uncover a role for EPHX3 in fatty acid epoxide hydrolysis, *Ephx3*^*-/-*^ mice can be useful in the future to investigate the potential role of EPHX3 in hydrolysis of other endogenous epoxide substrates and/or xenochemicals. This is especially important because selective EPHX3 inhibitors have not yet been developed.

The *Ephx3*^*-/-*^ mice did not exhibit an overt phenotype. There were no changes in body weight, organ weight, or reproductive capacity, and there were no gross anatomical abnormalities upon careful histologic review of internal organs. Both tissue lysates and plasma from *Ephx3*^*-/-*^ mice exhibit no differences in endogenous EET/DHET or EpOME/DiHOME ratios compared to WT mice. Moreover, lung, stomach, and skin lysates from *Ephx3*^*-/-*^ mice incubated with exogenous 8,9-EET, 11,12-EET, or 9,10-EpOME have similar diol formation rates as WT mice. Similar results were observed in incubations with both microsomal and cytosolic fractions from *Ephx3*^*-/-*^ mice. Together, these results indicate that *Ephx3*^*-/-*^ mice exhibit similar fatty acid metabolic profiles under basal conditions. We conclude that EPHX3 does not play a discernable role in fatty acid epoxide hydrolysis in the mouse, despite exhibiting high rates of metabolism of these epoxides *in vitro* [12].

Phenotypic differences can often be unmasked by *in vivo* challenges that perturb homeostasis. For example, *Ephx2*^-/-^ mice show no overt phenotype under basal conditions, but have altered responses in *in vivo* disease models. Therefore, we challenged *Ephx3*^-/-^ mice with cardiac ischemia/reperfusion injury and LPS-induced lung inflammation, which are both regulated by EETs [6,19]. No differences were observed in post-ischemic cardiac recovery between *Ephx3*^*-/-*^ and WT hearts. Moreover, although *EPHX3* is expressed in the lung at higher levels than either *EPHX1* or *EPHX2*, there was no difference between the *Ephx3*^*-/-*^ and WT mice with respect to LPS-induced lung inflammation. Importantly, the ratios of fatty acid epoxides:diols was not different after LPS treatment in *Ephx3*^*-/-*^ mice relative to WT mice. Overall, neither cardiac ischemia-injury nor LPS-induced inflammation unmasked phenotypes in the *Ephx3*^*-/-*^ mice.

The results from our *in vivo* metabolism studies are different than previously published *in vitro* studies that demonstrated that recombinant EPHX3 protein could metabolize various fatty acid epoxides, including EETs and EpOMEs. Several factors may account for these differences. First, the *in vitro* study was performed in a reconstituted system with the recombinant protein and single substrates, which may not necessarily mimic in vivo conditions. Second, it may also be that EPHX3 is only capable of EET and EpOME hydrolysis at high concentrations of fatty acid epoxides. The *in vitro* studies determined that EPHX3 has rapid turnover (*V*_*max*_ values were 10 times faster than EPHX2); however, it only achieved these rates at high concentrations (*K*_*m*_ values were 30–130 μM epoxides) [12]_._ The *in vivo* concentration of fatty acid substrates is likely to be in the low nM range, well below the *K*_*m*_ of EPHX3. EPHX1 or EPHX2 may be the predominant enzyme responsible for fatty acid epoxide hydrolysis at these lower substrate concentrations.

Numerous interactions may occur in our *in vivo* disease model that may mask the impact of *Ephx3* disruption. We may not observe changes in EET metabolism in the *Ephx3*^*-/-*^ mice because a large portion of EET hydrolysis may occur through EPHX1 or EPHX2 that is still present in the *Ephx3*^*-/-*^ mice. In this regard, we plan to generate mice deficient in both EPHX2 and EPHX3, both EPHX1 and EPHX3, and all three EPHX isoforms. This will allow us to determine if the fatty acid metabolism and phenotypes of the *Ephx1*^-/-^ and *Ephx2*^*-/-*^ mice are further altered by disruption of *Ephx3*, and also study the potential contributions of these these enzymes to fatty acid epoxide metabolism.

It remains possible that there are other, yet to be identified, substrates for EPHX3 *in vivo* that are unrelated to fatty acid epoxide hydrolysis. EPHX1 and EPHX2 have been shown to hydrolyze a variety of compounds that are unrelated to fatty acid epoxides [20]. EPHX1 readily hydrolyzes butadiene oxide, styrene oxide, benzo(α)pyrene oxide, and androstene oxide. Likewise, EPHX2 can hydrolyze phenyl-propene oxide and *trans*-stilbene oxide. EPHX1 is highly expressed in the liver and has a critical role in detoxification [20]. Interestingly, EPHX3 protein orthologs have been identified in many mammals and have relatively high sequence homology (>70% amino acid identity to human EHPX3), which suggests an important role for the conserved EPHX3 protein *in vivo*. Although EPHX3 expression in the liver is low, abundant EPHX3 expression in tissues exposed to environmental agents (skin, lung, stomach and intestine) does suggest a role for EPHX3 in xenobiotic metabolism. Limitations of *in vivo* animal experiments, coupled with the abundance of possible epoxygenated substrates, creates a difficult task to identify *in vivo* substrates for EPHX3. In this regard, we have begun a data-driven metabolomics approach to determine if other endogenous substances are altered in the *Ephx3*^*-/-*^ mice. A previous study suggested that EPHX3 may be a candidate gene for ichthyosis, a debilitting skin condition [21]. Moreover, several studies showed that CpG islands in the promoter of *Ephx3* are methylated in various cancer cell lines [22,23]. It may be that the role of EPHX3 will need to be explored in the context of skin disorders or cancer.

In summary, this study determined that EPHX3 does not play a significant role in the metabolism of epoxygenated fatty acids (including AA, LA, EPA or DHA) *in vivo*. In addition, *Ephx3* disruption did not alter LPS-induced lung inflammation or functional recovery after ischemia/reperfusion injury, two models known to be regulated by EETs and EPHX2. A continued search to identify the *in vivo* function of EPHX3 is important for several reasons. First, EPHX3 is highly conserved across multiple mammalian species which suggests an important physiological role. Second, the tissue distribution of EPHX3 expression is distinct from both EPHX1 and EPHX2. The abundance of EPHX3 in the skin, lung, stomach, and esophagus suggests an important role for this protein in xenobiotic detoxification in these tissues. Despite *in vitro* data that implies a role for EPHX3 in EET and EpOME hydrolysis, we conclude that EPHX3 has, at most, only a minor role in fatty acid epoxide hydrolysis *in vivo*.
